# Supplementary material for: MIPhy: identify and quantify rapidly evolving members of large gene families
Source: PeerJ. 2018 May 29;6:e4873. doi: 10.7717/peerj.4873 (PMC5983006; doi:10.7717/peerj.4873)
Supplement: Supplemental Information 1 — Table S1: The nematode collagens identified in this work from species in the genus Caenorhabditis. Article S1: The MIPhy model of gene family evolution. Figure S1: Example species and gene trees. [file peerj-06-4873-s001.docx]

Supporting information

**Table S1:** The nematode collagens identified in this work from species in the genus *Caenorhabditis*.

| **Species** | **# of collagen genes** |
| --- | --- |
| *C. angaria* | 127 |
| *C. brenneri* | 209 |
| *C. briggsae* | 173 |
| *C. elegans* | 181 |
| *C. japonica* | 197 |
| *C. remanei* | 145 |
| *C. sinica* | 163 |
| *C. tropicalis* | 155 |

# Article S1. The MIPhy model of gene family evolution

Here we present the MIPhy reconciliation and clustering algorithm. It proceeds in two phases, the first inferring the gene events and using them to generate an initial clustering, and the second phase incorporating traditional data clustering techniques to refine the clusters. The model of gene family evolution is derived from the core reconciliation methods of NOTUNG, with key modifications. That algorithm only allows incongruence (in the form of incomplete lineage sorting) at polytomies. Incongruence may appear due to errors in sequencing/gene-finding, incompletely resolved branches in tree-building software, horizontal gene transfer (HGT), or it may be due to selective pressures acting on one or more species. As such we freely include these events in our reconciliation. Moreover, we assume that incongruence is more likely than a duplication event followed by several independent loss events, so the latter case is not considered as possible history. As an example, node n6 in Fig. S1 is an incongruence event because genes a1 and b1 are closer to gene d1 than to c1, whereas the species tree suggests that a1 and b1 should be closer to gene c1 than to d1. If we did not allow incongruence events, n6 would instead be classified as a duplication, and the reconciliation would require three additional loss events.


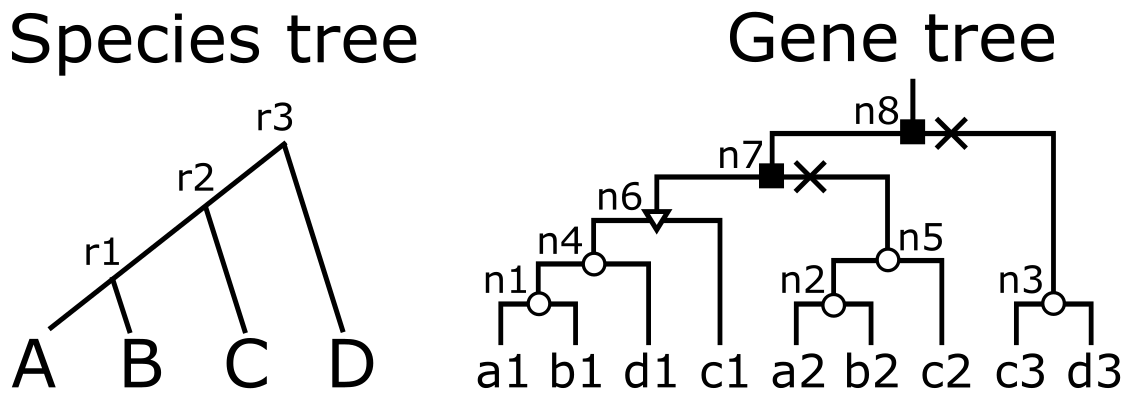


**Figure S1: Example species and gene trees.**

In the gene tree the gene events are indicated with filled squares, open circles, open triangles, and Xs, representing duplication, speciation, incongruence, and loss events, respectively. Nodes a1 and a2 in the gene tree represent two distinct genes from species A, b1 and b2 are genes from species B, and so on.

It should be noted that this, and many other parsimony algorithms, define a duplication to be the presence of at least one gene from the same species in both children of some tree node. This is not sufficient to rigorously prove that a duplication has taken place in some ancestral species, but this definition has been found to perform well in practice. Another difference compared to NOTUNG is that MIPhy does not attempt to model HGT explicitly. Instead, these events will be classified as incongruence or duplications, which both contribute to the phylogenetic instability cost function. This also allows the algorithm to classify these gene events using purely local information in a single pass through $T_{G}$, decreasing the time complexity by orders of magnitude.

## Terms and definitions

Given a gene tree $T_{G}$, let $g$ represent some node, where $l$ and $r$ are its children. If $g$ is a terminal leaf, its originating species $ori\left( g \right)$ is defined to be the species in the species tree $T_{S}$ from which the sequence $g$ was collected. If $g$ is an internal node, $ori\left( g \right)$ is defined to be the most recent common ancestor in $T_{S}$ of $ori\left( l \right)$ and $ori\left( r \right)$. The lineage of a node $lin(g)$ is the set of species nodes (including ancestral species) tracing $ori\left( g \right)$ back to the root of $T_{S}$. The set of all terminal leaves in the subtree of $T_{G}$ rooted by $g$ is given by $lvs\left( g \right)$. The species represented in the subtree of $T_{G}$ rooted at $g$, $spc(g)$, is the set obtained by applying $ori\left( c \right)$ to every leaf $c$ in $lvs\left( g \right)$; $S_{G}$ is the set of species in $T_{S}$ with at least one gene in $T_{G}$. The represented species of a node $g$ not present in the represented species of a node $h$ is given by $miss\left( g,h \right)=spc\left( g \right)-spc\left( h \right)$.

One of three mutually exclusive gene events must take place at each internal node in $T_{G}$: duplication, speciation, or incongruence. These are quantified by the binary variables $E_{D}\left( g \right)$, $E_{S}\left( g \right)$, and $E_{I}\left( g \right)$, respectively, constrained such that $E_{D}\left( g \right)+E_{S}\left( g \right)+E_{I}\left( g \right)=1$.

## Event inference

If $T_{S}$ and $T_{G}$ are given by the species and gene trees in Fig. S1, the gene events taking place at every internal node are inferred as follows:

- $E_{D}\left( g \right)=1 if spc\left( l \right)\cap spc\left( r \right)\neq\emptyset$: Node $g$ is a duplication event if its children share any represented species. As an example, $E_{D}\left( n7 \right)=1$ because $spc\left( n6 \right)=\left\{ A,B,C,D \right\}$ and $spc\left( n5 \right)=\left\{ A,B,C \right\}$, so $spc\left( n6 \right)\cap spc\left( n5 \right)=\left\{ A,B,C \right\}$.
- $E_{S}\left( g \right)=1 if E_{D}\left( g \right)=0 and ori\left( l \right)\notin lin\left( r \right)\wedge ori\left( r \right)\notin lin(l)$: Node $g$ is a speciation event if it is not a duplication event, and the originating species of neither child is contained in the lineage of the other. As an example, $E_{S}\left( n4 \right)=1$ because $E_{D}\left( n4 \right)=0$, $ori\left( n1 \right)\notin lin\left( d1 \right)$ $\left( r1\notin\left\{ D,r3 \right\} \right)$, and $ori\left( d1 \right)\notin lin\left( n1 \right)$ $(D\notin\left\{ r1,r2,r3 \right\}$.
- $E_{I}\left( g \right)=1 if E_{S}\left( g \right)+E_{I}\left( g \right)=0$: More explicitly, node $g$ is an incongruence event if it is not a duplication event, and the originating species of one child is contained in the lineage of the other. As an example, $E_{I}\left( n6 \right)=1$ because $E_{D}\left( n6 \right)=0$ and $ori\left( n4 \right)\in lin\left( c1 \right)$ $\left( r3\in\left\{ C,r2,r3 \right\} \right)$.

Minimum instability groups (MIGs) are defined by the most recent common ancestor in $T_{G}$ of the leaves in that group, and the numbers of duplication and incongruence events counted in the MIG defined by node $g$ are found by the recursive equations:

$$\begin{aligned} D\left( g \right)=E_{D}\left( g \right)+D\left( l \right)+D\left( r \right),\#\left( 1 \right) \end{aligned}$$

and $\begin{aligned} I\left( g \right)=E_{I}\left( g \right)+I\left( l \right)+I\left( r \right).\#\left( 2 \right) \end{aligned}$

A speciation event indicates that genes from one species (or ancestral species) will be found exclusively in the descendants of one child and not the other. Conversely, for both children of a duplication event node there should be one gene from every species that has not yet been excluded by a previous speciation or incongruence event. Loss events are therefore counted at duplication nodes, as the number of represented species of each child not present in the other:

$L^{'}\left( g \right)=E_{D}\left( g \right)\cdot loss\left( g \right)+L^{'}\left( l \right)+L^{'}\left( r \right)$,

where $loss\left( g \right)=\left| miss(l,r) \right|+\left| miss(r,l) \right|$.

This would only be accurate if every species is represented by at least one gene in the total species of each MIG. To complete this concept, we introduce a new quantity $M\left( g \right)$ that compares the represented species under $g$ with the total represented species $S_{G}$. Thus, the total loss events counted in the descendants of some node $g$ would be given by:

$$\begin{aligned} L\left( g \right)=L^{'}\left( g \right)+M\left( g \right),\#\left( 3 \right) \end{aligned}$$

where $M\left( g \right)=\left| S_{G}-spc\left( g \right) \right|$.

If $T_{G}$ is from Fig. S1, $L\left( n5 \right)=L^{'}\left( n5 \right)+M\left( n5 \right)=0+1=1$ because no genes from species D are present in $lvs\left( n5 \right)$, while $L\left( n7 \right)=L^{'}\left( n7 \right)+M\left( n7 \right)=1+0=1$. As demonstrated here, the $M$ term does not propagate up the tree, and tends to disappear as the algorithm progresses further from the leaves.

The above equations are somewhat naïve, as they do not allow for loss in ancestral species. If $T_{S}$ is given by Fig. S1 and we consider the MIG rooted by node n3, the above equations would calculate that two loss events have occurred, once each for species A and B. However, a more parsimonious explanation is that the ancestral homolog was only lost once, in species r1, the ancestor of A and B. We therefore redefine the constituents of equation (3) to account for these processes:

$L^{'}\left( g \right)=E_{D}\left( g \right)\cdot loss\left( g \right)+L^{'}\left( l \right)+L^{'}\left( r \right)$,

where $loss\left( g \right)=\max_{s\in spc(l)} \left| \left\{ ori\left( s,t \right) | t\in miss\left( r,l \right) \right\} \right|+\max_{s\in spc(r)} \left| \left\{ ori\left( s,t \right) | t\in miss\left( l,r \right) \right\} \right|$,

and $M\left( g \right)=\max_{s\in spc(g)} \left| \left\{ ori\left( s,t \right) \right|t\in\left\{ S_{G}-spc\left( g \right) \right\} \right\}|$.

An example from Fig. S1:

$$M\left( n3 \right)=\max\left( \left| \left\{ ori\left( C,A \right),ori\left( C,B \right) \right\} \right|, \left| \left\{ ori\left( D,A \right),ori\left( D,B \right) \right\} \right| \right)$$

$$M\left( n3 \right)=\max\left( \left| \left\{ r2 \right\} \right|, \left| \left\{ r3 \right\} \right| \right)=\max\left( 1,1 \right)=1$$

$$\therefore L\left( n3 \right)=L^{'}\left( n3 \right)+M\left( n3 \right)=0+1=1$$

## Initial clustering

This ‘Initial clustering’ section is also described in the main text, but is reproduced here for ease of reading. For a node $g$, $migs\left( g \right)$ is a set of sets describing the most parsimonious clustering pattern for those sequences in $lvs\left( g \right)$, where each inner set describes one MIG. These groups are built iteratively by comparing $sep\left( g \right)$, the score if the existing clustering patterns $migs\left( l \right)$ and $migs\left( r \right)$ are kept intact, with $cmb\left( g \right)$, the score if all descendants are combined into a single MIG; the minimum of these two values is stored as $best(g)$. After the initial clustering phase, the overall clustering pattern is described by $migs\left( root \right)$; every sequence from $T_{G}$ is contained exactly once in $migs\left( root \right)$. Worked examples of these variables can be found in Table S2.

The weighted sum of equations (1), (2), and (3) constitutes the score function:

$$\begin{aligned} score\left( g \right)=\theta_{D}\cdot D\left( g \right)+\theta_{I}\cdot I\left( g \right)+\theta_{L}\cdot L\left( g \right)+\theta_{P}\cdot P\left( g \right),\#\left( 4 \right) \end{aligned}$$

where the $\theta$ values are the strictly positive weights applied to each event, and $P(g)$ is a the “relative spread” metric defined by equation (5) in the next section; for this initial phase of the algorithm it is set to 0. Each node $g$ in $T_{G}$ is visited in a post-order depth-first traversal. The algorithm is described by the following pseudocode:

If $g$ is a terminal node:

$best\left( g \right)=M(g)$

$migs\left( g \right)=\{\left\{ g \right\}\}$

Otherwise:

$cmb\left( g \right)=score(g)$

$sep\left( g \right)=best\left( l \right)+best(r)$

If $cmb\left( g \right)\leq sep(g)$, all descendants of $g$ are merged into one MIG:

$best\left( g \right)=cmb(g)$

$migs\left( g \right)=\{lvs\left( g \right)\}$

Otherwise the existing cluster patterns of nodes $l$ and $r$ are kept intact:

$best\left( g \right)=sep(g)$

$migs\left( g \right)=migs(l)\cup migs(r)$

## Transforming phylogenetic distances to coordinate points with multi-dimensional scaling

The second phase of the MIPhy algorithm evaluates and refines the clusters generated by in the initial phase. Several metrics exist to measure the spread between points in a cluster compared to the rest of the data. However, many require that these points be embedded into a coordinate system, such as Euclidian space, having properties such as the concept of a mean. A phylogenetic tree does not possess these properties, so we use multi-dimensional scaling to transform the nodes of the tree into a set of coordinate points that respect the phylogenetic distances between each sequence.

First, the full pairwise distance matrix from $T_{G}$ is generated as the matrix $D$, such that $D_{ij}$ is the phylogenetic distance (measured as the sum of the branch lengths) between the leaves $i$ and $j$. The Gram matrix $M$ can then be generated by:

$$M_{ij}=\frac{D_{i1}^{2}+D_{1j}^{2}-D_{ij}^{2}}{2}$$

where ‘sequence 1’ is an arbitrary choice held constant throughout the calculation of the matrix (this sequence will be located at the origin of the coordinate system). The coordinate points can then be found by eigenvalue decomposition. If $M=USU^{T}$ is solved, the $i$th row of the matrix $X=U\sqrt{S}$ contains the coordinates for the point representing leaf $i$ from $T_{G}$.

## Cluster refinement

These coordinate points are used in the “relative spread” calculation:

$$\begin{aligned} P\left( g \right)=\frac{\sigma\left( g \right)}{\bar{\sigma}}-1,\#\left( 5 \right) \end{aligned}$$

where $\sigma(g)$ is the standard deviation of the points representing the sequences in the MIG rooted by $g$, and $\bar{\sigma}$ is the median standard deviation of all MIGs (excluding singleton clusters). The spread quantity is normalized around 0, so $P\left( g \right)=1.0$ indicates that the spread of MIG $g$ is 100% larger than the median spread, while $P\left( h \right)=-0.3$ indicates that the spread of MIG $h$ is 30% smaller than $\bar{\sigma}$. Though MIPhy currently measures spread using a simple standard deviation, clustering-specific methods like the Davies-Bouldin index or silhouette could be easily substituted. As in the initial clustering phase, each node $g$ in $T_{G}$ is again visited in turn. The clustering procedure is repeated, this time including the relative spread term in the full score function in equation (4).

**Table S2: Worked MIPhy example.**

This table provides the variables used in the Event inference and Initial clustering phases of the MIPhy algorithm applied to Fig. S1. Only one set of terminal leaves is included for brevity. The $best\left( g \right)$ value for each node isn’t explicitly stated, but is indicated by the bolded score value in either $cmb\left( g \right)$ or $sep\left( g \right)$. The values $\theta_{D}$, $\theta_{I}$, and $\theta_{L}$, indicate the weight of one duplication, incongruence, or loss event, respectively. The final clustering pattern is found at the root, $migs\left( n8 \right)$, and here the algorithm predicts three clusters. Interestingly, the clustering pattern for this tree is invariant for all parameter weights such that $\theta_{I}<3\theta_{L}$. This is because in the entire table, the $cmb\left( n6 \right)$ to $sep\left( n6 \right)$ comparison is the only one that is not invariant; as an example, $cmb\left( n5 \right)<sep\left( n5 \right)$ $\left( \theta_{L}<4\theta_{L} \right)$ is true for all strictly positive weights – which is true of these parameter weights by definition – as is $cmb\left( n7 \right)>sep\left( n7 \right)$ $\left( \theta_{D}+\theta_{I}+\theta_{L}>\theta_{I}+\theta_{L} \right)$.

|  | **Event inference values** | | | | | **Initial clustering values** | | |
| --- | --- | --- | --- | --- | --- | --- | --- | --- |
| $g$ | $ori(g)$ | $lin(g)$ | $lvs(g)$ | $spc(g)$ | Event$ent species tree,S, or loss event, respectivelynt inference and Initial clustering phases of the MIPhy algorithm. of the best$ | $cmb(g)$ | $sep(g)$ | $migs(g)$ |
| a1 | A | $\left\{ A,r1,r2,r3 \right\}$ | $\left\{ a1 \right\}$ | $\left\{ A \right\}$ | - | - | $\boldsymbol{3}\boldsymbol{\theta}_{\boldsymbol{L}}$ | $\left\{ \left\{ a1 \right\} \right\}$ |
| b1 | B | $\left\{ B,r1,r2,r3 \right\}$ | $\left\{ b1 \right\}$ | $\left\{ B \right\}$ | - | - | $\boldsymbol{3}\boldsymbol{\theta}_{\boldsymbol{L}}$ | $\left\{ \left\{ b1 \right\} \right\}$ |
| c1 | C | $\left\{ C,r2,r3 \right\}$ | $\left\{ c1 \right\}$ | $\left\{ C \right\}$ | - | - | $\boldsymbol{2}\boldsymbol{\theta}_{\boldsymbol{L}}$ | $\left\{ \left\{ c1 \right\} \right\}$ |
| d1 | D | $\left\{ D,r3 \right\}$ | $\left\{ d1 \right\}$ | $\left\{ D \right\}$ | - | - | $\boldsymbol{\theta}_{\boldsymbol{L}}$ | $\left\{ \left\{ d1 \right\} \right\}$ |
| n1 | r1 | $\left\{ r1,r2,r3 \right\}$ | $\left\{ a1,b1 \right\}$ | $\left\{ A,B \right\}$ | $E_{S}$ | $\boldsymbol{\theta}_{\boldsymbol{L}}$ | $6\theta_{L}$ | $\left\{ \left\{ a1,b1 \right\} \right\}$ |
| n2 | r1 | $\left\{ r1,r2,r3 \right\}$ | $\left\{ a2,b2 \right\}$ | $\left\{ A,B \right\}$ | $E_{S}$ | $\boldsymbol{2}\boldsymbol{\theta}_{\boldsymbol{L}}$ | $6\theta_{L}$ | $\left\{ \left\{ a2,b2 \right\} \right\}$ |
| n3 | r3 | $\left\{ r3 \right\}$ | $\left\{ c3,d3 \right\}$ | $\left\{ C,D \right\}$ | $E_{S}$ | $\boldsymbol{\theta}_{\boldsymbol{L}}$ | $3\theta_{L}$ | $\left\{ \left\{ c3,d3 \right\} \right\}$ |
| n4 | r3 | $\left\{ r3 \right\}$ | $\left\{ a1,b1,d1 \right\}$ | $\left\{ A,B,D \right\}$ | $E_{S}$ | $\boldsymbol{\theta}_{\boldsymbol{L}}$ | $3\theta_{L}$ | $\left\{ \left\{ a1,b1,d1 \right\} \right\}$ |
| n5 | r2 | $\left\{ r2,r3 \right\}$ | $\left\{ a2,b2,c2 \right\}$ | $\left\{ A,B,C \right\}$ | $E_{S}$ | $\boldsymbol{\theta}_{\boldsymbol{L}}$ | $4\theta_{L}$ | $\left\{ \left\{ a2,b2,c2 \right\} \right\}$ |
| n6 | r3 | $\left\{ r3 \right\}$ | $\left\{ \begin{aligned} a1,b1, \\ c1,d1 \end{aligned} \right\}$ | $\left\{ A,B,C,D \right\}$ | $E_{I}$ | $\boldsymbol{\theta}_{\boldsymbol{I}}$ | $3\theta_{L}$ | $\left\{ \left\{ a1,b1,c1,d1 \right\} \right\}$ |
| n7 | r3 | $\left\{ r3 \right\}$ | $\left\{ \begin{aligned} a1,b1, \\ c1,d1, \\ a2,b2,c2 \end{aligned} \right\}$ | $\left\{ A,B,C,D \right\}$ | $E_{D}$ | $\theta_{D}+\theta_{I}+\theta_{L}$ | $\boldsymbol{\theta}_{\boldsymbol{I}}\boldsymbol{+}\boldsymbol{\theta}_{\boldsymbol{L}}$ | $\left\{ \begin{aligned} \left\{ a1,b1,c1,d1 \right\}, \\ \left\{ a2,b2,c2 \right\} \end{aligned} \right\}$ |
| n8 | r3 | $\left\{ r3 \right\}$ | $\left\{ \begin{aligned} a1,b1,c1, \\ d1,a2,b2, \\ c2,c3,d3 \end{aligned} \right\}$ | $\left\{ A,B,C,D \right\}$ | $E_{D}$ | $2\theta_{D}+\theta_{I}+2\theta_{L}$ | $\boldsymbol{\theta}_{\boldsymbol{I}}\boldsymbol{+2}\boldsymbol{\theta}_{\boldsymbol{L}}$ | $\left\{ \begin{aligned} \left\{ a1,b1,c1,d1 \right\}, \\ \left\{ a2,b2,c2 \right\}, \\ \left\{ c3,d3 \right\} \end{aligned} \right\}$ |
